# Bio-tensegrity and extracellular matrix disruption in the pathogenesis of persistent post-operative pain: a hypothesis

**DOI:** 10.3389/fphys.2025.1647334

**Published:** 2025-09-09

**Authors:** Shiloh Plaut

**Affiliations:** ^1^ Department of General and Internal Medicine, Shaare Zedek Medical Center, Jerusalem, Israel; ^2^ Department of basic and clinical sciences, University of Nicosia, Nicosia, Cyprus

**Keywords:** connective tissue, extracellular matrix, fascia, fascial armoring, models theoretical, myofibroblasts, pain postoperative, psychosomatic medicine

## Abstract

Persistent post-operative pain (PPOP) is a significant and often debilitating outcome of invasive procedures, with prevalences ranging from 30% to 70% following certain types of surgery. Yet, despite high prevalences and ongoing rigorous research, the pathophysiological mechanisms underlying PPOP remain insufficiently understood. While neurobiological explanations such as nerve injury, peripheral and central sensitization, and neuroma formation have been proposed, theory-based treatments provide only limited relief, resulting in persisting pain and decreased quality of life for affected individuals. This paper presents a framework for the theoretical pathogenesis of PPOP based on a work published recently which offered a connective-tissue-based mechanobioneurological mechanism for the pathophysiology of functional psychosomatic pain syndromes (or “fibromyalgia-type syndromes”), framing fibromyalgia and PPOP as disorders whose mechanism is based in the extracellular matrix’s homeostasis drawing on continuum biomechanics. With its aetiology and mechanisms in dispute, fibromyalgia, which historically was regarded as a connective tissue disorder, has long been a medical mystery. The model offers a mechanistic explanation for ‘primary fibromyalgia syndrome’ as a non-autoimmune disease driven by overactivity of myofascial and interstitial myofibroblasts that sustain mechanical stress within the fascio-musculoskeletal system. Fascia is a hugely overlooked complex delicate viscoelastic and fiber-cellular tissue that extends throughout the human body at various depths and layers and constitutes a complex dynamic interconnected extensive network of connective tissue that undergoes a process of continuous remodeling and transmits and absorbs loads while exhibiting tensegrity-type qualities. Surgical interventions may disrupt biomechanical stability, inducing chronic pain and central neurophysiological aberrations. The model frames these conditions as disorders of interconnected neurobiological and biomechanical systems and opens a new avenue for research on the link between neurobiology and connective tissue.

## 1 Introduction

Chronic pain after surgical procedures is a major health concern, impacting the postsurgical outcome of patients, their ability to fully rehabilitate, and their quality of life. The World Health Organization’s International Classification of Diseases 11th edition defines persistent postoperative pain (PPOP) as pain lasting more than 3 months after surgery (i.e., chronic pain), in the absence of any other pain-causing factors such as infection or pre-existing comorbid conditions (Treede et al., 2015). Despite extensive advancements in surgical techniques, anesthesia, and pain management practices, the mechanisms that drive PPOP remain elusive. Several theoretical patho-mechanisms have been proposed to explain PPOP, with most focusing on neurobiology and psychology, such as neuroinflammation and nerve or vascular injury, biopsychosocial models, and long-term changes in synaptic structures that transpire as hypersensitivity to pain (i.e., peripheral and central sensitization) (Richebé et al., 2018). Table 1 shows estimated incidence rates of PPOP in different surgical operations as given in a review by Fuller et al. (2023). Persistent post-mastectomy pain is a notable example of an operative complication that can exert significant impact on one’s quality of life (Jung et al., 2003). Its prevalence is estimated to be above ∼30% in mastectomy patients and is mostly attributed to neuropathological causes (Jung et al., 2003; Tait et al., 2018). It remains unclear though why some individuals develop PPOP while others do not. Box 1 outlines the general current understanding of PPOP pathogenesis.

**TABLE 1:** Estimated incidence of persistent postsurgical pain as summarized in a review by Fuller et al. (2023).

Surgery type	Estimated incidence of persistent postsurgical pain (%)
Thoracotomy	5–71
Amputation	30–60
Coronary bypass	30–50
Sternotomy	7–17
Cholecystectomy	3–56
Hip arthroplasty	7–23
Knee arthroplasty	13–14
Inguinal herniotomy	5–63
Dental surgery	5–13
Craniotomy	7–30
Mastectomy	11–57
Caesarean section	6–55

There are other chronic pain disorders whose underlying mechanisms are undeciphered; one example is the phenomenon of fibromyalgia syndrome, and for it, too, the scientific community focuses mainly on neurobiological explanations. Nevertheless, while the theories mainly try to explain hyperalgesia and pain rather than the syndrome, there are certain gaps in empirical studies that neuroscience theories, as they are currently framed, seem to have difficulty bridging (Adkisson et al., 2014; Goebel et al., 2021; Holdcroft and Jaggar, 2005; Plaut, 2022). Examples of such gaps are findings of autoantibodies, discrepancy between subjective disease burden and pressure pain threshold, poor success of current theory-based treatments, migrating multisite pain, tender spots, explaining what role small fibre pathology has, and more.

Fibromyalgia, the “queen” of chronic pain, is a controversial chronic pain condition of unknown aetiology and poorly understood pathophysiology, that typically manifests as widespread musculoskeletal pain, low mood, post exertional malaise, and chronic fatigue, affecting at least ∼2–6 percent of the general adult population, but prevalence estimates differ according to definitions and methodologies used in epidemiological studies. Fibromyalgia leads to a significant burden on the healthcare system and considerably impacts patients’ quality of life and psychological wellbeing (Holdcroft and Jaggar, 2005).

In the 19th century a surgeon named Dr. William Balfour described fibromyalgia (historically termed “rheumatism,” and subsequently “fibrositis”) as a disease of connective tissue, but because no adequate theoretical mechanism was offered to explain its various symptoms and anomalies for decades it remained a medical enigma. It was only afterwards in the 1990s that a new hypothesis was proposed claiming fibromyalgia to be a neurological disease of the central nervous system, and it soon gained favor. In the absence of a convicting peripheral pathology to explain the syndrome, a neurological origin seemed much more sensible (see, for example, Simms, 1996), mainly because there seemed to be a strong mental component to the syndrome and the treatments are often of the antidepressants or gabapentenoids class of medications. Fibromyalgia patients often experience ‘brain fog’, clumsiness, autonomic symptoms, sleep disturbances, and co-occurring psychiatric disorders of low mood, hypervigilance, distress, and anxiety. Although its exact mechanisms remain unclear too, “central sensitization” and dysregulation of ascending and descending pain processing pathways in the peripheral nervous system, dorsal root ganglion, spinal cord dorsal horn, and brain, are currently the most widely accepted explanations and the most investigated paradigm (Adkisson et al., 2014; Goebel et al., 2021; Holdcroft and Jaggar, 2005). Despite intense research, numerous anomalies and counterinstances persist and the patho-mechanisms of fibromyalgia continue to be poorly understood, and the field remains in relative stagnation in terms of translation to therapeutic clinical impact, partly because ambiguity in the theory hinders the development of effective treatments and identifying specific targets for disease modifying interventions. “Fibromyalgia” as a clinical phenomenon is still so bewildering that many clinicians think it is a made-up concept–a “non-disease” - or attribute it to depression, despite not being all that common in those with major depression disorder (Holdcroft and Jaggar, 2005). With no cure, treatment focuses on symptomatic management by a combination of pharmacotherapies, physical therapy, lifestyle modification, and psychological support (Adkisson et al., 2014; Holdcroft and Jaggar, 2005).

A recent study meanwhile offered a theoretical model with an organic neuro-mechanobiological mechanism to help explain the pathogenesis of functional psychosomatic “fibromyalgia-type” syndromes and myofascial pain syndromes, describing these as clinical variations of a non-autoimmune connective tissue disease driven by inflammatory myofibroblasts in soft tissue and interstitium (Plaut, 2022). The term ‘psychosomatic syndromes’ within the context of this paper refers to disorders that are usually attributed to mental, emotional, or psychological disorders manifesting somatically in the body top-down (e.g., via neuroendocrine pathways) without tissue histopathological abnormalities, typically regarded in medicine as a subjective experience, categorized as non-life-threatening psychological-distress-induced disorders of organ functionality, not true organic diseases, i.e., disorders of function, not of tissue integrity, composition, architecture, or structure. In terms of nosology, what distinguishes primary fibromyalgia syndrome from other functional (psycho)somatic syndromes is simply a matter of fashion of clinical definition, since the “diagnosis” is not biologically attached to a specific measurable mechanism. Thus, from the standpoint of molecular biology, what truly distinguishes between these clinical syndromes is still not entirely understood. Fibromyalgia is one of the psychosomatic syndromes. The purpose of this paper is to apply this model to PPOP.

Box 1 **General overview of PPOP pathogenesis.**

It is accepted that inflammatory responses and nerve injury cause long-term synaptic plasticity in psychologically and genetically prone individuals, which then maintains and amplifies pain signaling and hyperalgesia, a process called “pain sensitization”. A more elaborate review on PPOP and its pathophysiology is available in the work of Richebé et al. (2018). So far studies in animal models have demonstrated that post-incisional nociception produces cellular and biochemical responses that are distinct from other pain models. Essentially, invasive surgery initiates nociceptive stimuli that lead to biomolecular changes in local tissue such as release of nerve growth factor and cytokines, alongside alterations in the primary sensory neurons of the dorsal root ganglia and epigenetic changes in the spinal cord. Incision into the skin and muscle during surgery triggers a series of transcriptional level changes in the primary afferent sensory neurons of the dorsal root ganglia, persisting long after the operation. These changes affect nociceptor activation and affect processes like tissue remodeling, nerve regeneration, wound healing, and the immune system response. Empirical studies suggest that neuroplastic changes in the central nervous system occur secondary to and together with postoperative peripheral sensitization. Excitatory neurotransmitters, notably glutamate and substance P, as well as activation of glial cells, are implicated as key mediators in these processes. Additionally, descending pain inhibitory systems and endogenous analgesia may eventually become downregulated or exhausted in PPOP, further amplifying pain (Richebé et al., 2018). This thesis usually guides PPOP’s treatment plan.

## 2 Hypothesis and conceptual framework

Building on the previously proposed theoretical model for fibromyalgia pathogenesis, in which myofibroblast hyperactivity and fascial mechanical compression were suggested as potential contributors to the manifestation of fibromyalgia-type psychosomatic pain (Plaut, 2022), a key hypothesis that’s derived from the model posits that surgical interventions that modulate the fascio-musculoskeletal tensegrity-like system will significantly impact fibromyalgia-type conditions. Empirical findings from studies in the clinical setting indicate that, for an unclear reason, certain surgeries can completely relieve fibromyalgia symptoms while other types of surgeries trigger or worsen fibromyalgia (Adkisson et al., 2014; Costantini et al., 2016; Plaut, 2022; Saber et al., 2008).

Adkisson et al. (2014) documented the complete resolution of fibromyalgia symptoms after parathyroidectomy. Saber et al. (2008) describe complete resolution of fibromyalgia after laparoscopic Roux-en-Y surgery, and Costantini et al. (2016) describe significant changes in fibromyalgia disease trajectories after laparoscopic cholecystectomy. The reasons for such marked changes in fibromyalgia’s disease course due to surgery are still unclear.

The model also suggests that invasive procedures might lead to the development of new-onset persisting postsurgical pain due to changes in the myofascial bio-tensegrity system and thus prompt myofascial and neuropathic pain. ‘Bio-tensegrity’ denotes the dynamic behavior of a living system that is stabilized by compressive and tensile force elements, a characteristic property attributed to myofascial tissue (Chaitow et al., 2012) - the extracellular matrix being the main medium for continuum biomechanics here. Once the extracellular matrix is remodeled around the sensory nerve fibres in a state of higher substrate rigidity, the new altered microenvironment would predispose to spontaneous nociceptive signal firing due to mechanical and chemical stimuli, mediated by soft tissue myofibroblasts. In this work the theoretical model of Fascial Armouring will be presented in an attempt to explain the mechanism of persistent postsurgical pain as a disorder of neuro-fascio-musculo-skeletal origin. Elucidating pathophysiology is crucial for advancing disease modifying therapeutic strategies beyond symptomatic management.

The attributed shared role of central sensitization in the pathogenesis of both PPOP and fibromyalgia is a recurrent theme in contemporary scientific literature (Fuller et al., 2023), an acknowledgement that these two phenomena might involve a shared mechanism. The following section will present the theoretical model of fascial armouring that was developed for fibromyalgia and applies it to PPOP.

## 3 The theoretical model of fascial armouring to help explain fibromyalgia-type manifestations and persistent postoperative pain

### 3.1 Fascial armouring

The theoretical model of “Fascial Armouring” describes how the upregulation of myofibroblast phenotype cells in myofascial tissue can help explain various medically unexplained manifestations of “primary fibromyalgia syndrome,” and related functional somatic syndromes, as variations of one medical entity of rheumato-psycho-neurology (Plaut, 2022). Functional somatic syndromes are suggested to be phenotypic variations of one medical entity that shares a common mechanism (Chen et al., 2021; Wessely et al., 1999). “Fascial armouring” offers a mechanobioneurological organic mechanism and an explicit biological pathway for the pathogenesis of fibromyalgia-type syndromes, that’s based on the following five elements (this section presents the conceptual framework and theoretical model for the pathogenesis of fibromyalgia, and, afterwards, in section 3.2., the clinical implications will be discussed):

#### 3.1.1 Fascia

**FIGURE 1:**
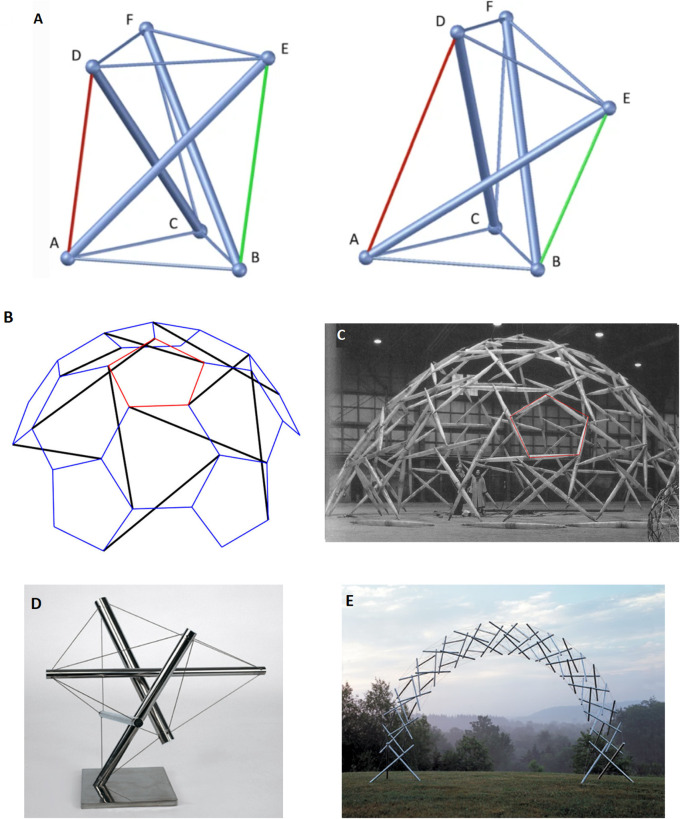
An illustration of the concept of tensegrity. Its aim is to illustrate an anatomical situation of mechanical imbalance in the (fascio)musculoskeletal system. The purpose is to demonstrate tensegrity as a pillar in the model, not a specific clinical syndrome. Floating compression elements transfer force through the tension elements. Changes to one node affect mechanical homeostasis of the structure and other nodes as well. Returning from our allegory back to the living human body, internal mechanical forces may be long-lasting (e.g., sustained by tissue remodeling, tissue fibrosis, and tissue contracture) or acute (e.g., myofibroblast myosin light chain kinase mediated contractions induced by mechanical stimuli such as palpation). The clinical manifestation *de facto* depends on the anatomical area, the visceral structures involved, density of innervation, *etc.*, and even psychological factors. **(a)** A simple free-standing three-dimensional tensegrity structure in a symmetric (left) and a non-symmetric (right) configuration. The non-symmetric state occurs as the green cable is shortened while the red cable is lengthened. Figure from Micheletti and Podio-Guidugli (2022), under open access license permission. **(b)** Tensegrity sphere model in schematic geometry, and **(c)** model structure as realized by Buckminster Fuller. Depicts a simplified model for us, representing the (fascio)musculoskeletal system, although technically speaking a prestressed system with floating compression elements may be more representative of a human body. Mechanical forces are dispersed in multiple directions and travel in the system through myofascial chains and affect anatomical structures and interstitium. Of importance are embedded nerve fibers. Any shift or imbalance in the structure caused by a surgical intervention may potentially manifest clinically. Figure from Micheletti and Podio-Guidugli (2022). **(d)** a “bio-tensegrity structure” leaning towards the reader’s right side, which may reflect imbalance and cause symptoms such as pain (60.5° 1992 stainless steel tensegrity structure by Kenneth Snelson http://kennethsnelson.net). **(e)** is a symmetrical arch tensegrity structure representing a balanced system as a simplified model of the bio-tensegrity system (Rainbow arch 2001 by Kenneth Snelson image from http://kennethsnelson.net). But suppose you cut one cable with a scalpel.

The first building block for the model is the continuity and interconnectedness of fascia in conjunction with tensegrity qualities: Fascia is a fundamental component of the fascio-musculo-skeletal system, forming a dynamic, body-wide fiber-cellular and viscoelastic connective tissue network that provides support, shape, and stability. It extends across various layers and depths, integrating and enveloping muscles and anatomical structures, viscera, and interstitum, surrounding, permeating, and connecting epimysia, perimysia, tendons, ligaments, retinacula, septa, aponeuroses, blood vessels, epineuria, periostea, and connective tissue sheaths at various depths and layers, while exhibiting mechanical qualities corresponding to tensegrity (or ‘bio-tensegrity’) (Chaitow et al., 2012; Tadeo et al., 2014). ‘Tensegrity’ is an architectural term that refers to the homeostasis of a complex structure that can be stabilized under dynamic forces of compression and tension, and functions as one connected system (Micheletti and Podio-Guidugli, 2022; Tadeo et al., 2014). ‘Bio-tensegrity’ is a conceptual framework that applies the principles of tensegrity to better understand the biomechanics and physiology in living organisms. It integrates biology of living systems into a tensegral-biophysical framework under continuum biomechanics, where each “individual component” is considered in relation to the whole system (Chaitow et al., 2012; Scarr, 2020). Figures 1A–C below display tensegrity structures as an illustration. As shown, changes applied in one part of the system translate into a change in the overall state of the entire system and the balance of forces within it. The figure was brought to illustrate an anatomical situation of mechanical imbalance in the (fascio)musculoskeletal system. The purpose is to demonstrate tensegrity as a pillar in the model, not a specific clinical syndrome.

The pervasiveness and interconnectedness of fascia has been observed, for example, by the intramuscular fascia being continuous with collagen-reinforced structures such as neurovascular tracts, compartmental fasciae, intermuscular septa, and interosseous membranes (Wilke et al., 2018). The endomysium and muscle fibers fuse at the ends and along the full peripheral length of the muscle fiber (Wilke et al., 2018). Myofascial tissue even has physical contiguity with the meninges through the myodural bridge (Zheng et al., 2017).

In living tissues there’s an ongoing dynamic balance of forces of cell traction and points of resistance within the extracellular matrix (ECM), with a state of reciprocal isometric mechanical tension. The dynamic bio-tensegral system and mechano-transducing signaling enable cells to mechanically sense changes, modify their microenvironment, and promote ECM remodeling in homeostasis and in disease states (Tadeo et al., 2014). Observational studies highlighted the relevance of bio-tensegrity mechanotransduction on tumor cells by mediating the cellular response to ECM stiffness (López-Carrasco et al., 2020; Tadeo et al., 2014). In addition, existing empirical investigations of ECM (and fascia) in humans *in vivo* support the tensegrity properties of fascia by demonstrating its role in a continuous myofascial system where tension is balanced across different segments. For example, studies have shown that sustained manual pressure on the lateral raphe in patients with chronic low back pain resulted in an anterior shift of the transversus abdominis musculofascial corset system, suggesting the release of pre-existing tightness or adhesion in the posterior fascia and a change in its elastic properties (Chen et al., 2016). Manual intervention has also been shown to lead to increased sliding and thickness changes of the transversus abdominis, indicating a redistribution of tension within the myofascial system (Chen et al., 2016). Furthermore, research on isometric plantar-flexion demonstrated a strong correlation in stiffness changes between the lower limb muscles (gastrocnemius) and lumbar tissues (thoracolumbar fascia and erector spinae), highlighting a long-distance interaction within the myofascial tensegrity network (Chen et al., 2022). These findings collectively reinforce the concept of fascia as a force transduction network rather than merely local passive structures, supporting its tensegrity role in maintaining body stability and function. Virtually all organs and tissues are organized as prestressed structural hierarchies that exhibit immediate mechanical responsiveness and increase their stiffness in direct proportion to the applied mechanical stress (Ingber, 2006). Molecules, cells, tissues, organs, and our entire bodies use “tensegrity” architecture to mechanically stabilize their shape, and to harmonize structure and function at all size scales (Ingber, 2008).

#### 3.1.2 Myofibroblasts

Myofibroblasts are cells normally found in fascia and maintain basal mechanical tissue tone (Chaitow et al., 2012; Fede et al., 2021). These native cells possess contractile activity and mechanosensitive regulation, with a positive feedback loop of force generation, which allows them to lock in tension in the ECM with significant amount of force (Tomasek et al., 2002). Focal adhesion complexes are protein structures that enable myofibroblasts to bridge the cytoskeleton with the surrounding matrix by serving as a mechanical link (Tomasek et al., 2002) and in addition transmit downstream signals and recruit more biomolecular elements in a force dependent manner (Tadeo et al., 2014). By synthesizing alpha smooth muscle actin and then incorporating stress fibres within the cells, in conjunction with continuous ECM remodeling, myofibroblasts simultaneously stress-shield themselves from external mechanical stress while maintaining tissue contracture in their surrounding ECM (Tomasek et al., 2002). Their mechano-activity is facilitated in large by transforming growth factor beta 1 and Rho-associated kinase signaling pathways (Chaitow et al., 2012; Martin et al., 2016). Tryptophan and serotonin are probably also utilized as part of the normal function of myofibroblasts biology and feedback regulations (Dolivo et al., 2018). Several authors have suggested, based on calculations, that the contractile forces generated by myofibroblasts might be substantial enough to impact the dynamics of the musculoskeletal system (Schleip and Klingler, 2019). It was estimated that forces generated by soft tissue myofibroblasts may reach ∼2 N and generate 1 cm per month of contracture that’s sustained by matrix remodeling, which is not at all negligible (Schleip and Klingler, 2019; Wilke et al., 2018). Myofascial compression and matrix contractility, caused by myofibroblast generated bio-tensegrity tensional pre-stress and ECM remodeling, may help explain several of fibromyalgia’s manifestations (such as pain, distribution of pain, trigger/tender points, hypersensitivity to mechanical pressure, hyperalgesia, allodynia, chronic fatigue, metabolic abnormalities and oxidative stress, autonomic abnormalities, cardiovascular abnormalities, morning stiffness, small fiber pathology, various psychosomatic symptoms, close association with hypermobility syndrome, lack of overt inflammation, silent imaging investigations, and other phenomena such as complete resolution soon after surgery) (Plaut, 2022). Myofibroblasts generally have similar activity irrespective of their anatomical location (Majno et al., 1971), although the “fibroblast” cell type is a diverse family of cells (Holl et al., 2024; Stecco et al., 2018; Xie et al., 2018). More than one protein conformations of αα-SMA exist, and not all myofibroblasts express α-SMA (Tomasek et al., 2002) so using it as a marker for fibrogenic cell activity in skeletal muscle or other tissues may be problematic (Sun et al., 2016; Xie et al., 2018).

#### 3.1.3 Myofascial chains

Fascia is not a uniform tissue but a complex three-dimensional web of dense and loose connective tissues that transmits forces to a distance by means of mechanical links called myofascial chains (Chaitow et al., 2012; Wilke et al., 2016; Wilke and Krause, 2019). Internal forces created by muscles are transferred and dispersed to neighboring and more distant structures in the body, such that, for example, mechanical tension in the plantar aponeurosis can affect the lumbar fascia/erector spinae muscle (through the gastrocnemius muscle/fascia, hamstring muscles/fasciae, sacrotuberous ligament, *etc.*) (Wilke et al., 2016; 2018). The classic outdated perception in medicine that skeletal muscles and their ligamentous links are isolated independent actuators that are comparable to a robot or a puppet on strings needs a thorough revision (Wilke et al., 2018).

#### 3.1.4 Fibroblasts/myofibroblasts as a network

Langevin and colleagues (Langevin et al., 2004) have shown, using confocal microscopy, histochemistry, immunohistochemistry and electron microscopy, that cultured fibroblasts of mouse subcutaneous tissue as well as cultured human fibroblasts form a widespread interconnected cellular network with potentially substantial physiological importance. About 30% of such processes could be followed continuously from one cell to another using confocal microscopy. Myofibroblasts are present in fascia of normal healthy individuals, they help maintain basal mechanical tissue tone (Fede et al., 2021; Schleip and Klingler, 2019) and function as a large network (Hansson and Skiöldebrand, 2015). Fibroblasts/myofibroblasts can couple via gap junctions and function as a syncytium or electrically coupled network (Andoh et al., 2007; Grafstein et al., 2000; Mercier and Hatton, 2000). Given the capacity of myofibroblasts for intercellular coupling via gap junctions (Follonier et al., 2008; Tomasek et al., 2002) which makes them physiologically relevant in cardiac arrythmias (Nguyen et al., 2012) and bladder dysfunctions (Sui et al., 2008), and as they have stretch dependent calcium channels (Arora et al., 1994) and are able to generate synchronized contractions through the transfer of calcium waves (Castella et al., 2010), myofibroblast contraction and intracellular calcium are suggested to be mechanistically linked (Castella et al., 2010).

#### 3.1.5 Somatosensation

Fascia houses an abundant network of sensory nerve endings that are involved in nociception and pain perception (Fede et al., 2020; Sinhorim et al., 2021). Pathological changes in fascia are characterized by increased tissue stiffness and changes in the extracellular matrix, including changes in both collagens and matrix metalloproteinases levels as well as alteration in myofibroblast activity (Fede et al., 2020). Myofascial tissue is richly innervated and contains proprioceptors, mechanoreceptors, and nociceptors, and sympathetic fibers, thus being involved in the development of pain (Chaitow et al., 2012; Suarez-rodriguez et al., 2022). Abnormal mechanical forces and nociceptive mediators that are secreted by myofibroblasts and local cells (e.g., interleukin 1-beta, tumor necrosis factor-alpha, neuropeptide Y, substance P) may trigger pain via activation of peripheral sensory receptors (Chaitow et al., 2012). It is suggested that in myofascial pain syndrome, the local extracellular milieu is acidic and has elevated levels of cytokines and neurotransmitters, such as interleukin-6, substance P, bradykinin, and calcitonin-gene-related-peptide indicating hypoxic conditions in the muscle (Shah et al., 2005) which is also relevant for nociception. This acidic environment contributes to inhibition of acetylcholine esterase and therefore to an elevated concentration of acetylcholine molecules at the motor endplate, which is of relevance for myofascial trigger points and myofascial pain (Gerwin, 2023). Researchers have investigated the effect of substrate matrix rigidity on neuronal cells *in vitro*, and found a marked difference in growth dynamics, synaptic density and electrophysiological activity of cortical neuronal networks when comparing cultures grown in substrates with 100-fold differences of young modulus (Lantoine et al., 2016). The pre-synaptic density was two times higher on stiff substrates and consistently the number of action potentials and miniature synaptic currents was enhanced on stiff substrates (Lantoine et al., 2016).

### 3.2 Assembling the building blocks into a theoretical model

These abovementioned findings in five building blocks are integrated to form the theoretical model of “fascial armouring,” based on a neuro-mechanobiological mechanism that can be summarized as: myofibroblast-generated bio-tensegrity tension, ECM rigidity, mechanical compression, and subsequent corresponding neurophysiological aberrations, on a background of interrelated myofascial tissue and myofascial chains. It is essentially a myofibroblast-mediated disease of biophysical tensegrity. In its severe form, this mechanism, committed to stiffness of ECM, is expected to manifest clinically as a sort of mild-to-moderate global chronic exertional compartment-like syndrome, with longstanding low-grade hypoxia and systemic oxidative stress, deep muscle ache and discomfort, new unfamiliar spinal and bodily stiffness, absence of overt systemic inflammation, post-exertional malaise, mild pallor, and physiological compensatory secretion of kynurenine, bradykinin, as well as nitric oxide to dilate blood vessels and improve perfusion. This which may help explain various manifestations of “fibromyalgia” such as (i) myalgia, myofascial discomfort, and myofascial pain, (ii) distribution of pain, (iii) trigger points/tender spots, (iv) chronic fatigue, (v) cardiovascular abnormalities, (vi) metabolic alterations, (vii) autonomic symptoms, (viii) morning stiffness and altered gait, (ix) small fibre pathology, (x) various psychosomatic symptoms, (xi) close association with hypermobility syndrome, (xii) lack of obvious systemic or local inflammation, (xiii) silent routine medical investigations, and other phenomena such as occasional complete resolution soon after surgery (Plaut, 2022).

Thus, when clinically interpreting this entity under the model of fascial armouring using clinical reasoning, for instance, one may infer from the mechanism that if myofibroblast mediated bio-tensegrity tension and fascial stiffness develop in or adjacent to the temporal fascia, it is expected to manifest as a tension-type “primary headache disorder”. If bio-tensegrity imbalance and compression are transmitted to the thoracolumbar fascia, in conjunction with myofibroblast ECM remodeling, the expected manifestation would be a chronic “non-specific” low back pain. If “fascial armouring” affects myofascial tissue in the head, jaw, shoulders, and neck area and the continuous myofascial channels associated with them, it would manifest as “non-specific” neck pain. Torso or chest-shallow breathing, chest tightness, and if severe, “non-specific” non-cardiac chest pain. Pelvic fascia-genitourinary symptoms and myofascial pelvic pain. Cervical fascia and compression of the superior cervical ganglion-dry mouth and sicca. Compression of sympathetic nerves, solar plexus and abdomen/diaphragm-autonomic abnormalities and asynchrony between breathing rhythm as directed by the medulla and the movement of the diaphragm. Mesentery and gut wall-disturbances in gut peristalsis and impaired bowel function. Baroreceptors-blood pressure dysregulation and postural orthostatic intolerance. Stellate ganglion-sympathetic imbalance. Proprioceptors-imbalance, impaired coordination, and microsomatognosia. Joint capsule or tendons and mild contractures-decreased joint range of motion, and if myotatic reflex is involved-muscle spasms. Cutaneous and subcutaneous ECM alterations-easy bruising, pruritus, and small fiber pathology (Plaut, 2022). Arm-predisposition to carpal tunnel syndrome. Palms-cold palms and Raynaud’s-like phenomenon. Hair follicles-hair loss. Neck and pretracheal fascia-muscle tension dysphonia and dysphagia, and so forth. The guiding theme while clinically interpretating this entity is myofascial tension, compression, and ECM stiffness. If tension becomes diffuse and widespread, it is expected to present clinically with lassitude, mild pallor, decreased flexibility, mild change in handwriting, general feeling of heaviness, morning stiffness, irritability, and general myofascial discomfort and pain. Abnormal pendulousness affects gait and kinesthetics (e.g., new onset clumsiness). Altered absorption and dissipation of mechanical forces/energy in myofascial tissue would contribute to a “functional” disorder of movement without classic neurological signs. Unrefreshing sleep is a non-specific symptom that can easily be attributed to psychological issues. This mechanism obviously has inherent variability so the clinical manifestation would depend on multiple factors such as the anatomical structures involved, layers of fascia, taut bands, genetic factors, compensatory mechanisms, psychological factors, *etc.* Furthermore, unmyelinated muscle nociceptors are activated by hypoxia of muscle tissue which is exacerbated by muscle contraction (Mense and Stahnke, 1983; Staud, 2011).

The following presents empirical findings from studies that, when combined, may support this fascia-based mechanism for fibromyalgia as an entity resembling a global chronic exertional compartment-like syndrome:

1) A recent study found that intramuscular pressure in trapezius muscle of fibromyalgia patients is significantly higher than normal with a mean pressure of 33 mmHg compared to 12 mmHg in rheumatic disease controls (Katz et al., 2021). The pressure abnormality in muscle of fibromyalgia patients may potentially underly the diffuse muscle pain and explain the overall clinical picture of the disease. Thus, the idea of central sensitization as being the sole mechanism of fibromyalgia should be revisited (Katz et al., 2021). It is recognized that in many cases symptoms attributed to “central sensitization” are actually maintained by bottom-up sensory signals originating in peripheral tissue such as soft tissue and the musculoskeletal system (Staud, 2011). Studies measuring intramuscular pressure of other muscles besides trapezius in fibromyalgia were not found.
2) The local pain of chronic widespread pain and fibromyalgia patients is often related to the presence of myofascial trigger points (Staud, 2011). For the most part, myofascial trigger points make up the topography of fibromyalgia tender spots (r = 0.78, p-value <0.001) (Ge et al., 2010).
3) Significantly higher measurements of muscle damping were found in fibromyalgia patients which reflects increased muscle tension (Wachter et al., 1996). Myofascial taut bands (i.e., palpable hardened and contracted muscle fibers) are common in fibromyalgia patients (Wolfe et al., 1992). According to a patient survey, morning stiffness was ranked as being bothersome to a similar degree as pain in individuals suffering from fibromyalgia (Bennett et al., 2007).
4) Findings from biopsy studies in fibromyalgia using electron microscopy showed segmental muscle fiber necrosis with lipid and glycogen deposition as well as subsarcolemmal mitochondrial accumulation (Kalyan-Raman et al., 1984). These were found in all sampled cases and were suggested to be the result of sustained muscle contraction and ischemia of unknown aetiology (Kalyan-Raman et al., 1984). This is a non-specific finding indicative of long-standing hypoxia in skeletal muscle.
5) Peripheral fibroblasts from fibromyalgia patients express significantly higher levels of TGF-β (Evdokimov et al., 2020).
6) The decreased threshold for pain seen in fibromyalgia may be due to collagen crosslinking abnormalities which may contribute to ECM remodeling and collagen deposition around the nerve fibers (Sprott et al., 1997) Fibromyalgia patients were found to have a significantly lower amount of intramuscular collagen, potentially explaining a predisposition for muscle micro-injury and may underly findings of non-specific muscle pathology (Gronemann et al., 2004).
7) Investigations with microdialysis in fibromyalgia revealed higher concentrations of interstitial metabolic substances including lactate, glutamate, and pyruvate (Gerdle et al., 2010).
8) A reduction in peripheral blood flow occurs in individuals with fibromyalgia, which suggests the presence of functional disruptions within the cardiovascular system (Morf et al., 2005). The blood flow of muscles seems to be reduced in fibromyalgia (Elvin et al., 2006; Staud, 2011).
9) Fibromyalgia patients exhibit an altered hypomethylation pattern in DNA, specifically in genes that are involved in stress response, free radical clearance, and DNA repair (Ciampi de Andrade et al., 2017). Oxidative stress seems to have an important role in fibromyalgia’s clinical picture (La Rubia et al., 2013).

Taken together, these findings might suggest an underlying myofascial abnormality in fibromyalgia syndrome. A more elaborated analysis of this mechanism in the context of fibromyalgia and psychosomatic syndromes can be found in a recent study (Plaut, 2022). To illustrate the model of Fascial Armoring, Figures 1D,E depict a tensegrity structure as a simplification of this framework, representing the human body, where mechanical forces are able to travel in the system through mechanical links. Tensing fascia in one area of the system allows for the transmission of mechanical forces throughout the connected nodes of the structure. Any shift or imbalance in the pre-stressed tensegrity structure may lead to clinical manifestations, depending on the area, depth, force, and anatomical structures involved. Surgery is expected to cause a sudden shift and pathological imbalance in the bio-tensegral homeostasis and affect the biophysical microenvironment of local peripheral nerve fibers.

### 3.3 Summarizing the model of “fascial armoring"

(i) A conceptual framework is presented for the pathogenesis of fibromyalgia syndrome stemming from aberrant activity of connective tissue myofibroblast cells that, when analyzed from a perspective of continuum biomechanics and tensegrity, can help explain various medically unexplained manifestations of the syndrome, besides hyperalgesia and idiopathic pain. This framework can be summarized as myofibroblast-mediated bio-tensegrity tension, ECM disarray, and mechanical compression on a background of interrelated myofascial tissue and myofascial chains. As a disease, this mechanism can be portrayed as a sort of “mild-to-moderate global chronic compartment-like syndrome” (Plaut, 2022).
(ii) TGF-β and mechano-transduction signaling pathways of focal adhesion complexes in fibroblast phenotype cells and inflammatory myofibroblasts are at the core of the suggested mechanism ultimately driving a diffuse widespread myofascial tissue transformation.
(iii) The tensegrity-like qualities of the myofascioskeletal system are exhibited by the pervasiveness of ECM and dispersion and transmission of tensional forces by means of anatomical connections of muscle-tendinous-fascial tissue, which is at the basis of this theoretical model.

### 3.4 Surgery in the framework of biophysical tensegrity

Beyond the acute effects of tissue injury, surgery can have long lasting effects on fascia. Notably, scar tissue contains a high number of myofibroblasts (Henderson et al., 2020). These cells create and transmit mechanical force to the surrounding ECM making use of myosin light chain kinase and rho kinase mediated signaling pathways. In the context of PPOP pathogenesis, neuromas may form whenever peripheral nerves are injured (Jung et al., 2003). A neuroma consists of tangled axons that are unable to regenerate to their target, as well as fibroblasts and other cells. Chronic neuropathic pain after surgery is often attributed to the formation of a neuroma in scar tissue. For instance, in the context of mastectomy, iatrogenic damage and scarring may create an environment for neuromas to form, and axons encased within these scars can lead to spontaneous pain and heightened mechanosensitivity (Jung et al., 2003).

Myofibroblast cells, which are seen in scar tissue following surgery, generate mechanical and chemical stimuli in the local ECM which can initiate nociception, resulting in pain and sensations in myofascial tissues without other ongoing active injury (Chaitow et al., 2012). Myofibroblasts are a phenotype of mechano-active smooth-muscle-like cell which generally have a similar behavior and mechanobiology irrespective of the anatomical location or the tissue (Majno et al., 1971). In such a scenario where inflammatory myofibroblasts are overactivated in peripheral tissue or interstitium, the presence of a nearby neuroma would allow for input of more complex sensations being itself a sensitive apparatus able to send afferent signals in response to mechanical and biochemical stimuli.

It is known that a neuroma can serve as a source of ectopic discharges and afferent input to the spinal cord, driving spontaneous pain (Flor et al., 2006). The neuroma has intrinsic hyperexcitability (e.g., due to inflammatory milieu, ion channel dysregulation, ectopic electrogenesis) and is not merely a passive actor. Interestingly, myofibroblasts appear to have a role in neuroma pathobiology (Badalamente et al., 1985). In a microscopy and biochemical study of human neuromas, myofibroblasts were found in samples of patients, along with a dense glycosaminoglycan matrix. Myofibroblast were associated to each other and to surrounding collagen. Sampled nerves from control subjects on the other hand showed rather normal ultrastructural components such as myelinated and unmyelinated neurofibres, characteristically surrounded by Schwann cells, but without myofibroblasts (Badalamente et al., 1985). It is, therefore, not unreasonable to suggest that the presence of myofibroblasts in neuromas may contribute to neuroma ectopic signals and hyperexcitability.

Evidence for the crucial role of ECM stiffness in nerve cell function, which underpins this suggestion, is found in data that ECM stiffness is a crucial factor in the behavior and function of nerve cells (Kayal et al., 2020). Matrix rigidity exerts significant effects on neuronal cells showing a marked difference in growth dynamics, synaptic density and electrophysiological activity of cortical neuronal networks when comparing cultures grown in substrates with 100-fold differences of young modulus (Lantoine et al., 2016). Matrix stiffness is a significant parameter that modulates Schwann cell function and behavior (Gu et al., 2012).

**FIGURE 2:**
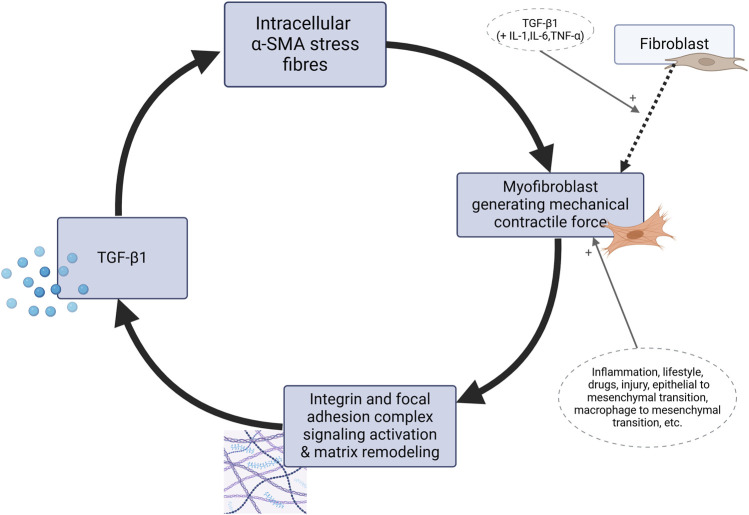
The positive feedback loop of myofibroblasts as the basis for the mechanism of “fascial armoring”. Matrix metalloproteinases, ECM material, cytokines, chemokines, growth factors and neurotrophins including nerve growth-factor are secreted by myofibroblasts in a copious secretory profile but the focus of this diagram is on the mechanobiological cascade involving TGF-β, without disregarding constituents like interleukin-1-beta, interleukin 6, connective tissue growth factor, platelet derived growth factor, tumor necrosis factor alpha, ED-A fibronectin, *etc.* TGF-β is secreted in a latent form to the ECM (Tomasek et al., 2002) and activated through interaction with integrins, not shown here for the purpose of simplicity. The intermediate proto-myofibroblast phenotype is not shown. TGF, transforming growth factor. SMA, smooth muscle actin. IL, interleukin. TNF, tumor necrosis factor.

To summarize, Figure 2 outlines the self-perpetuating loop of myofibroblast contractile activity which transpires as TGF-β enhances mechanical contractile activity of myofibroblasts while its levels are also sustained by mechanical stress. The figure explicitly shows the core molecular pathway of the mechanism. Its clinical significance is the outcome of the whole mechanism. Various factors provide stimulating or suppressing input into the cycle, claiming a potential role in aetiology. This process, when occurring in myofascial tissue resident myofibroblasts, was postulated to be the basic cellular mechanism to drive syndromes of “central sensitization syndromes” and “primary fibromyalgia syndrome” (Plaut, 2023). In this framework, myofibroblasts within fascia are thus suggested to significantly influence the ECM tensegrity and contribute to psychosomatic pain and PPOP through several key mechanisms:

1. The increased tension, shear forces, and stiffness of ECM caused by myofibroblast activity directly stimulate mechanosensitive nociceptors or polymodal fibres within the myofascial tissue. These nociceptors respond to mechanical deformation and pressure/stretch/traction, firing signals that are interpreted as pain once processed in the brain. Also, in certain instances, the altered mechanical environment can lower their threshold for activation, making them more easily triggered. Findings of Lantoine et al., 2016 provide the biological basis for a plausible link between ECM rigidity and pain manifesting clinically.
2. Excessive collagen deposition and tissue remodeling associated with myofibroblast activity can potentially lead to the entrapment or compression of small nerve fibers within the deep or superficial fascia.
3. The effects of higher substrate rigidity on Schwann cells add another neuropathic component to the mechanism.
4. Under conditions of higher matrix stiffness, long term transcriptional and epigenetic level adaptations in nerve cells lead to changes in their structure and function, including intracellular cytoskeleton architecture, which can affect neuronal electrophysiology.
5. Myofibroblasts can release various pro-inflammatory mediators and growth factors that can sensitize nociceptors in the surrounding tissue, lowering their threshold for activation by mechanical or chemical stimuli. This can lead to hyperalgesia (increased sensitivity to pain) and allodynia (pain from normally non-painful stimuli).
6. Local hypoxic conditions in muscle caused by epimysial and perimysial compression can lead to the release of algogenic substances from the affected tissue, and to the activation of chemoreceptors on nociceptors, and low-grade inflammation.
7. Myofibroblasts can communicate with neighboring cells, including other fibroblasts, myocytes, and cells of the nervous system via gap junctions. This allows for the direct transfer of electrical and chemical signals, potentially contributing electrophysiological input to the propagation of pain signals or the sensitization of nociceptors.
8. Changes in the mechanical properties of fascia due to myofibroblasts can affect proprioception and kinaesthesia. This altered sensory feedback can lead to compensatory movements, muscle imbalances, and increased strain on other tissues, which can indirectly contribute to pain.
9. Compression of the dorsal root ganglion and sympathetic chains potentially lower thresholds for electrophysiological activity and can even cause them to fire signals spontaneously. Increased rigidity and compressive forces in the ECM microenvironment may constitute a sufficient endogenous stimulus to independently initiate this phenomenon, in the absence of external stimuli.

This paper proposes a biomechanical model for PPOP, shifting focus from neurocentric theories to dysregulation of the fascial ECM and myofibroblast-mediated disruption of bio-tensegrity. The central hypothesis posits that surgery disrupts the body’s physiological tensegrity equilibrium, leading to abnormal ECM and altered remodeling by hyperactive myofibroblasts. ECM rigidity creates heightened mechanical tension and compression on sensory nerves and/or neuromas, generating nociceptive signals perceived as PPOP. The manuscript integrates concepts from connective tissue biology, mechano-transduction, and clinical observations (e.g., fibromyalgia, plantar fasciitis complications) to support its framework. Its main strength lies in proposing a fresh, integrative perspective on PPOP pathogenesis that challenges prevailing neurocentric models and highlights understudied peripheral mechanisms.

The presence of myofibroblasts specifically within neuromas potentially carries significant practical implications for understanding and treating neuropathic pain. Their contractile activity and ability to remodel the ECM could generate abnormal, sustained mechanical stress directly upon the sensitive nerve fibers within the neuroma. This mechanical stress, alongside their paracrine secretion of cytokines, could serve as a direct, localized source of nociceptor activation and sensitization. Consequently, targeting myofibroblast activity or modifying the stiffened ECM around neuromas represents a novel therapeutic strategy for alleviating intractable neuroma pain.

Finally, it is known that fascia and the tissues of the locomotor system have several layers and subdivisions as defined in textbooks, and that the classification to layers comes more from an anatomical standpoint. In reality, the extracellular matrix and fascial connective tissue is integrated as a connected network in the human body with interrelated and intermeshed tissues that are difficult to determine where one section ends and the next one begins, while they are all in relationship with one another functionally (Chaitow et al., 2012; Scarr, 2020). Therefore, the theoretical model presented here approaches fascia from this standpoint, as a connected tissue network. To explain the model, there is no need to focus on a specific layer as classified in the textbook.

## 4 Evaluation of the hypothesis: examples of new onset chronic pain following invasive interventions for exploring a framework of “bio-tensegrity”

In this section empirical findings are presented for illustrating the “bio-tensegrity” model of Fascial Armouring. The guiding theme while interpreting the following examples and anomalies is that any intervention that affects fascial tissue may cause a shift in bio-tensegrity force dynamics and can therefore cause pain and other symptoms adjacent or more distantly, due to abnormal mechanical forces in myofascial tissue which are sensed by sensory receptors.

• Plantar fascia rupture: In a study of 37 individuals with plantar fasciitis treated by corticosteroid injection into the calcaneal origin of the fascia (Sellman, 1994), 30% of patients experienced a sudden tearing sensation in the heel, while others had more gradual symptom changes. Though many found relief from heel pain, some patients experienced new problems such as pain in the metatarsals, midfoot (dorsal and lateral) discomfort, foot weakness, swelling, and even metatarsal fracture. Plantar fascia rupture occurred in all cases. While for most individuals the new symptoms improved within a year, for others the symptoms persisted.
• Another study encountered the same issue. While some patients with plantar fasciitis experienced a sudden rupture associated with a corticosteroid injection, others had a gradual onset of symptoms (Acevedo and Beskin, 1998). Despite achieving relief of the original pain, new troubles soon occurred such as lateral plantar nerve dysfunction, longitudinal arch strain, midfoot strain, hammertoe deformity, and even stress fracture. Diminished tension of the plantar fascia was demonstrated on examination by stretch test in all such patients (Acevedo and Beskin, 1998). At an average 27-month follow-up, 50% had good or excellent scores and 50% had fair or poor scores. Ten feet were asymptomatic by 6 months post rupture, four feet by 12 months post rupture, and 26 feet remained symptomatic 1 year post rupture.

According to the bio-tensegrity framework, this invasive treatment modality altered the fascia and therefore the entirety of the bio-tensegrity system. Complications occur, in this framework, due to the release of forces in fascia in areas of very high tension with a sudden change in the tensegrity’s state (Plaut, 2022). A sudden mechanical alteration can shift pre-existing forces to other areas and exacerbate imbalances. Steroid injection was shown to decrease fibromatosis and myofibroblasts in adhesive capsulitis (Hettrich et al., 2016), which suggests that injection to the plantar fascia may alter myofibroblast generated tensegrity forces (Plaut, 2022). It is possible that tension rather than entrapment causes the nerve dysfunction following plantar fascia rupture (Acevedo and Beskin, 1998).

Like any other model, the tensegrity model described here is a simplification of reality. There is evidence for fascial functional and structural continuity between the foot, leg and back (Behm et al., 2016; Chen et al., 2022; Wilke et al., 2016; Wilke et al., 2018) In addition, it was found that when plantar fascia rupture occurs, other anatomical and physiological anomalies occur (Sellman, 1994). When these two evidences are combined one can infer the presence of tensegrity type dynamics. It is worth noting that the fascial armoring model does not make it mandatory for the whole body to be involved in a specific situation.

Because tensegrities are composed of discrete networks of support elements, rather than a uniform medium like a chunk of metal or a rubber band, they provide a way to transmit mechanical forces along specific paths and to focus or concentrate stresses on distant sites and at different size scales. These are all features observed at the level of whole organs as well as tissues, cells, membranes, cytoskeletal networks, subcellular organelles, nuclei, mitotic spindles, transport vesicles, viruses, and proteins (Ingber, 2006). Clearly, post-injection complications (e.g., metatarsal pain, nerve dysfunction) may not be solely the consequences of bio-tensegrity disruption, and other potential mechanisms directly related to steroid injection may be involved (e.g., tissue weakening, altered biomechanics, direct neurotoxicity). However, a difficulty arises, since any explanation that relies on the effects of steroids, and any non-tensegrity explanation, leaves other anomalies unsolved, as discussed in the following.

• Phantom tooth pain has been described as such: *“following a tooth extraction, a pain behind the ear and on the side of the face in the day or so prior to facial weakness often constitute the earliest symptom of Bell’s palsy”* (Jameson et al., 2018. pp. 222–223). According to the bio-tensegrity model, the pain that develops behind the ear represents a reciprocal tensegrity “node” for the jaw. As an area containing curved bones and therefore susceptible to experiencing fascial biomechanical tension, the ear is anatomically adjacent to the emergence of the trigeminal and facial nerve from their canal. A new onset pain behind the ear indicates, according to the bio-tensegrity framework, new extreme tension - a tension like every other myofascial tension (e.g., plantar fasciitis). Invasive interventions in the face are expected to have a similar mechanical effect as does a calcaneal injection. If extreme tension is present in the pre-stressed tensegrity system, there will be enough potential elastic energy to damage structures in the face when released, as implied by a metatarsal fracture in the previous example listed above. In the face it causes a nerve palsy, not so different from that observed in the foot after plantar fascia rupture. If true, it seems there are two possible meanings for this palsy: either this palsy is not a Bell’s palsy, or a Bell’s palsy is not idiopathic. The reference (Jameson et al., 2018 pp.222–223) describes the phenomenon which is the anomaly. Our discussion here discusses the theory. It is true that the link drawn here between tooth extraction, ear pain, and Bell’s palsy as evidence for tensegrity disruption is highly speculative and not explored so far, therefore there is no literature on it, which calls for empirical studies to test it. The aim of this discussion is to discuss a mechanism for the anomaly. Obviously, since this Bell’s Palsy is an anomaly, an idiopathic condition, there is not much convincing evidence supporting any known mechanism so far. Hence, any pathophysiological connection remains speculative at this point. The discussion has not claimed that tensegrity is the state-of-art in Bell’s Palsy pathogenesis. It merely acknowledges that bio-tensegrity can predict these anomalies, and might help explain them. As with any other model, the tensegrity model described here is a simplification of the reality. The purpose of a theoretical model is to simplify, explain, and predict. Anyway, modulation of tensegrity force vectors might help us explain reported cases where:
• Carpal tunnel release increases the likelihood of developing trigger finger (Lin et al., 2017).
• Boutonniere deformity occurs following treatment of Dupuytren’s disease (Sanjuan-Cerveró et al., 2017).
• Treatment for lateral epicondylitis necessitates a supposedly unrelated shoulder arthroscopic decompression (Mishra et al., 2014).
• Simultaneous bilateral digital flexor ruptures occur (Gottlieb and Riskin, 1980).
• Compartment syndrome of the foot is diagnosed following spine surgery (Stotts et al., 2003).

These findings are not surprising under a bio-tensegrity framework since fascia is part of a continuous myofascial system with complex biomechanical properties. That fascia is more than mere passive collagen and functions as a dynamic and sophisticated tissue has been thoroughly substantiated by rigorous investigations of Schleip and colleagues (Chaitow et al., 2012; Schleip and Klingler, 2019) and Wilke and colleagues (Wilke et al., 2018). Connective tissue directly links most skeletal muscles in the human body (Wilke et al., 2016; 2017), and fascia is continuous from the trunk across the upper and lower limbs (Nordez et al., 2017). Acute bout of stretching of the upper limbs increases maximal range of motion of the distant lower limbs and *vice versa* (Behm et al., 2016). Significant changes in the stiffness of lumbar soft tissue and gastrocnemius occur with isometric plantar flexion, as shown by shear wave elastography measurements; this relationship is explained by it being a part of the myofascial tensegrity system (Chen et al., 2022). Unilateral stretching of one leg increases the range of motion of the contralateral leg, which is explained by the involvement of continuous structures such as the myofascias and the peripheral nervous system that form a link between the lower limb and the spine (Nordez et al., 2017). The neck, head, and eyes are arguably linked by anatomical myofascial continuum (Prabu Raja et al., 2022). Muscle and fascial tissues are interconnected, forming a myofascial network that functions in concert to facilitate the body’s movements with all parts of the body acting together as a whole (Chen et al., 2022).

## 5 Discussion

### 5.1 PPOP in light of the “bio-tensegrity” concept

The progression from acute to chronic postoperative pain is assumed to involve a complex interplay of biological, psychological, and socioenvironmental factors, but still remains a poorly understood process (Katz and Seltzer, 2009). The prevalence of PPOP varies across surgical procedures, with estimates ranging from ∼10% to ∼80% depending on the type of surgery, patient population studied, the methodology used, and other relevant factors (Ephraim et al., 2005; Richebé et al., 2018; Tait et al., 2018). PPOP’s impact is far-reaching, encompassing not only physical discomfort but also psychological distress, diminished quality of life, medicolegal aspects, and increased healthcare costs. The complexity of PPOP arises from its multifaceted nature, involving neurobiological, immunological, genetic and epigentic, psychological, and sociocultural factors. This complexity demands a comprehensive understanding of the underlying mechanisms to hopefully develop more effective treatments and prevention strategies.

This manuscript proposes a shift from purely neurocentric models of PPOP to a framework where connective tissue, specifically the extracellular matrix and inflammatory myofibroblasts, plays a central role in its pathogenesis. This offers a new perspective on a condition with poorly understood mechanisms. The paper introduces a model in which surgical interventions disrupt the biomechanical stability of the fascio-musculoskeletal system, acting as a key factor in inducing chronic pain. This highlights a unique biomechanical approach to understanding PPOP. By linking PPOP to an alternative connective-tissue-based paradigm for fibromyalgia, the manuscript suggests a broader, unifying mechanism for idiopathic and non-specific chronic musculoskeletal pain conditions. This connection opens new avenues for research into yet unexplored pathophysiological pathways and potential therapeutic targets. Bio-tensegrity is a useful framework that can help explain osteomyofascia phenomena and anomalies. Box 2 below summarizes the main points of the model.

Box 2 **Recapitulating the main points for the suggested model.**

**FIGURE 3:**
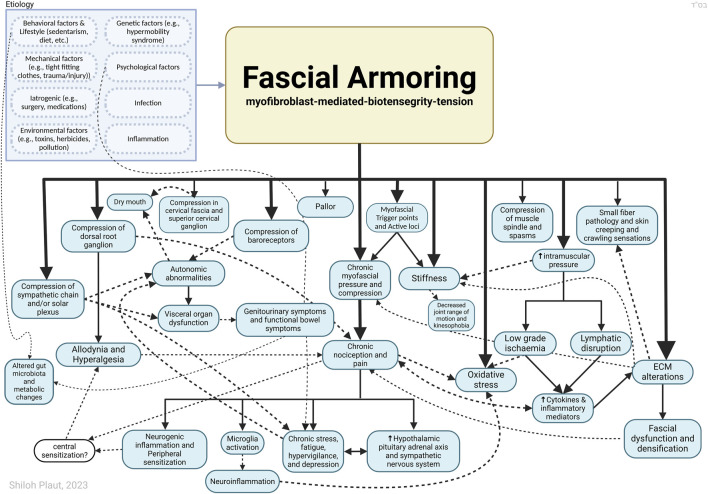
Outlining Fascial Armouring as a neuro-biomechanical medical entity in the form of bio-tensegrity tension and mechanical imbalance and compression, driven by myofibroblast-mediated bio-tensegrity-tension and extracellular matrix abnormalities. Not all relationships are depicted with arrows in this scheme. If the imbalance occurs more in a certain area, it may manifest as a local fibromyalgia-like disorder, primarily causing local pain. Altered deformation of fascia means altered transmission of mechanical loads. A seemingly arbitrary conglomerate of psychosomatic symptoms manifests as “fibromyalgia” when this pathology becomes widespread, resembling a sort of chronic exertional compartment-like syndrome of the whole body. Dashed arrows are meant for more visual clarity and have no special meaning. The insula is likely to be deeply involved in the psycho-emotional aspects of the disease as well as other brain regions (amygdala, prefrontal cortex, hippocampus, anterior cingulate cortex, *etc.*). The downstream effects of chronic pain and stress and activation of the hypothalamic-pituitary-adrenal axis have been well investigated by neurobiology and are beyond the scope of this diagram. Sympathetic nerves embedded within fascia provide a potential interface for psychology and fascial biomechanics. ECM, extracellular matrix. Figure created with BioRender.com adapted from “flow chart” (2023), retrieved from https://app.biorender.com/biorender-templates.

While the pathophysiological mechanisms of PPOP and fibromyalgia remain unclear, it is accepted that peripheral tissue and persistent nociceptive inputs have a crucial role in these chronic pain conditions and may drive them (Flor et al., 2006; Fuller et al., 2023; Katz et al., 2021; Richebé et al., 2018; Staud, 2011). The biophysical concept of bio-tensegrity provides a comprehensive view of the fascio-musculo-skeletal system where mechanical forces act not in isolation but affect the system as a whole. In this framework changes in one part of the system, for example, due to invasive procedures, may affect other parts of the system and potentially have long-lasting clinical implications (Figure 1 demonstrates the concept of tensegrity). Myofibroblasts in fascia can disrupt the delicate balance of the bio-tensegrity system by increasing tension and stiffness. This mechanical alteration, along with the release of sensitizing chemicals, can directly and indirectly activate nociceptors, leading to the perception of pain in idiopathic chronic musculoskeletal pain conditions. Bio-tensegrity, and a pathological disorder of it, can help explain various phenomena, and might be relevant in chronic pain states such as refractory post-operative pain, and is at the basis of the mechanism suggested here for fibromyalgia (in short: myofibroblast-generated bio-tensegrity tension, compression, and ECM stiffness). This is owing to fascia being a sensitive and sophisticated tissue that houses sensory and sympathetic nerve fibers and because pathological processes and dysfunction of fascia are suggested to promote chronic pain conditions. A myo/fibroblast dupuytren-like cord or tangled nodule, if found anywhere, could lock-in severe tension into the system, with clinical manifestations in clusters and patterns depending on the structures and chains involved. The sections above delineated a theoretical model and mechanism to offer a possible explanation for persistent pain in medically unexplained cases. Figures 2, 3 summarize the key points relating to the mechanism.

It is well-established that pain after surgery is not only due to inflammation or isolated nerve injury, but typically involves both (Richebé et al., 2018). Inflammation in conjunction with nerve injury may result in sustained signaling from peripheral nerves that activate nociception, to drive synaptic changes in the dorsal root ganglion and central nervous system, in a process that is mediated by various cytokines and neurotransmitters, with glutamate, brain-derived neurotrophic factor, and glial signaling being highlighted as main protagonists. The effect of mechanical strain and compression on peripheral nerve fibers, as well as local hypoxic conditions and inflammatory mediators, growth factors, and neurotrophins, secreted by myofibroblasts and local cells, are also relevant for chronic nociception and neuropathic pain in the suggested bio-tensegrity-based model.

Figure 3 illustrates “fascial armouring” as an entity of rheumato-psycho-neurology, driven by myofibroblast-generated tensegrity tension and compression and the downstream neurophysiological consequences of such. As shown, the aetiology is multifactorial and is also affected by lifestyle factors, specifically factors that promote myofibroblasts. A focal disease would manifest as a myofascial pain or “local fibromyalgia”. In cases where the condition becomes severe and widespread (i.e., “primary fibromyalgia syndrome”), the manifestation would resemble a mild-to-moderate global chronic compartment-like syndrome (Plaut, 2022). This may provide insight into “central sensitization symptoms,” though a comprehensive analysis of “fibromyalgia syndrome” falls outside the scope of this manuscript.

### 5.2 “All in the head”?

Although many cases of chronic postsurgical pain are believed to be of musculoskeletal origin (Tait et al., 2018), persistent pain following surgery is typically viewed as the result of a neuropathic and inflammatory process or a result of nerve injury during surgery (Richebé et al., 2018). The theoretical model presented in this work might help explain a subset of cases of PPOP, for example, the psychosomatic-type cases (i.e., in “non-specific” chronic fibromyalgia-like cases where no clear organic cause can be found for explaining the severity and character of patient-reported complaints, like pain and sensations, either at the site of surgery or more distantly). The model suggests a potential mechanistic link between fibromyalgia-type pain conditions and myofascial myofibroblasts. While empirical studies have not yet directly investigated this proposed link, some existing evidence supports a myofascial contribution to the condition, as discussed previously. Stecco and Fede et al. (Fede et al., 2020) examined the potential key role of fascial tissue in the development of postoperative pain. Post-surgical pain following total hip arthroplasty is said to be typically caused by fixation failure, instability, and soft tissue damage resulting from the surgical procedure. Magnetic resonance investigations have demonstrated a link between residual pain and soft tissue damage in the hip (Fede et al., 2020).

Tensegrity-based treatment modalities are expected to be beneficial in fibromyalgia and chronic postsurgical pain syndromes when targeting myofascial tissue correctly. Done incorrectly, an intervention would exacerbate patient-experienced pain. The potential efficacy of tensegrity-based treatments for PPOP is uncertain. Early evidence suggests that manual therapy based on tensegrity principles may improve postmastectomy pain by aiming to relieve the tension in surrounding muscles and soft tissues that are in direct and indirect contact with the affected area (Wilk et al., 2015). According to some authors, trigger point injection may be a useful treatment for persistent postmastectomy pain (Khoury et al., 2021). Soft tissue and fascia therefore should not be overlooked when diagnosing and treating PPOP. One must be careful not to jump to the conclusion that every pain in a distressed and perhaps otherwise healthy patient, that has no clear inflammation and no visible lesion to explain their nociception (and is refractory to analgesia and face-to-face reassurance) has “psychosomatic” pain or is cursed by central sensitization. Patients with persistent post-mastectomy pain often report that their symptoms are ignored, not taken seriously, or blamed on mental health issues (Jung et al., 2003; Khoury et al., 2021). This is a major obstacle for treatment for fibromyalgia patients as well.

The concept of ‘nociplastic pain’ aligns with the now mainstream notion that certain forms of chronic pain are better understood as conditions or diseases of their own, i.e., “pain as a disease” instead of “pain as a symptom.” And even though “central-sensitization” and “nociplastic/primary pain” tries to explain pain that arises in the absence of any actual or threatened tissue damage (or any pathology that can cause the justifiable activation of peripheral nociceptors) and in the absence of any evidence for a disease or lesion of the somatosensory system causing the pain (Kosek et al., 2021), this “all in the head” explanation is unsatisfactory (Katz et al., 2021; Plaut, 2022). Viewing fibromyalgia as a disease that is the misfortunate fate of traumatized and stressed neurotic individuals that have a genetic and psychological predisposition for an infinite positive feedback of pain with no real legitimate cause for nociception is misleading. It stigmatizes patients, and hinders their rehabilitation process, and lessens their trust in us as healthcare professionals. Brazenor et al. (2022) in a comprehensive literature review found no convincing evidence showing that central sensitization can persist as an autonomous pain generator after the initiating injury has healed.

Finally, even though bio-tensegrity is the conceptual framework of the model presented in this work, the model does not depend on an actual tensegrity relationship of the entire human body or myofascioskeletal system but rather relies on the existence of myofascial chains and force transmission to adjacent anatomical structures which may suffice for discussing “fascial armouring.” Tensegrity is integrated into the model as a simplification for the purpose of the discussion. Biomolecular and fluid dynamics of the soft tissue and hyaluronic acid produce a more complex system than plain mechanics of tensegrity. As with most models, its aim is to simplify.

### 5.3 Advantages of the fascial armoring model as a disease of rheumato-psycho-neurology

Compared to “central sensitization” and other neurocentric or psychosocial-based arguments made for fibromyalgia or medically unexplained chronic musculoskeletal pain, the neuromechanobiological model of “fascial armoring” has four important strengths:

(i) Fascia and ECM are present almost everywhere throughout the body, which means fascial armoring can (theoretically) affect any area of the body. Myofascial tissue is widely interconnected and is even contiguous with the meninges (Zheng et al., 2017). Also, cells have mechanosensitivity and substrate stiffness gradient guided migration as well as chemotaxis, meaning, this model is in no way limited only to myofascial tissue.
(ii) Fascia can apply significant mechanical forces, whereas many other medical entities cannot. Furthermore, the bio-tensegrity qualities of fascia suggest that an abnormality in a specific anatomical location is not limited to that area and may lead to clinically evident mechanical and non-mechanical effects at more distant seemingly unrelated sites (and can remain unmindful of dermatomes). Theoretically, mechanical forces exerted chronically on the dura could exert considerable physiological effects on the central nervous system. Studies have demonstrated that mechanical stress increases the levels of proteins tau, amyloid beta, and alpha-synuclein in brain of wild-type mice (Levy Nogueira et al., 2018). Evidence from *in vivo* research indicates that proprioceptor-induced microglial activation could be crucial for initiating and maintaining abnormal pain in individuals with chronic fatigue syndrome (Yasui et al., 2019).
(iii) Myo/fibroblasts are a diverse cell family. They secrete cytokines, growth factors, and various inflammatory mediators (Henderson et al., 2020), neurotrophins (Palazzo et al., 2012), and matrix metalloproteinases, and can uptake cellular signaling molecules and serotonin which affect molecular biological pathways and metabolism (Dolivo et al., 2018; Henderson et al., 2020; Tomasek et al., 2002). The cytokine profile can be distinct from neuronal profiles or from those of inflammatory diseases which are driven by leukocyte-related mechanisms. Systemic circulating inflammatory mediators are likely to affect intracranial ECM and the brain if allowed to cross the blood brain-barrier. The brain’s ECM is being investigated for its significance in neurological and psychiatric disorders (Dityatev et al., 2021; Sun et al., 2021).
(iv) Myofibroblasts can electrically couple to various types of cells via gap junctions (connexin 43) and function as one mechano-electrical network (Ikeda et al., 2007; Plaut, 2022). Studies have found that the meninges fibroblasts and brain cortex cells can electrically couple and may function as one large network (Grafstein et al., 2000; Mercier and Hatton, 2000). The mental psychological counterpart for this network’s mechano-electrical activity, if there is one, is so far unexplored. If myo/fibroblasts couple to the nervous systems and glial cells, providing external afferent input into the nervous system via this mechano-electrical fiber-cellular network might have an important psychological significance. The myo/fibroblast network of cells has patterns of contraction and these cells can transfer calcium waves that allow them to contract in collaboration (Arora et al., 1994; Castella et al., 2010; Plaut, 2022). Fascia is also in intimate relationship with sympathetic nerve fibers (Chaitow et al., 2012). Theoretically, a fascinating connection of mind-body could be formed in this model if the network of myo/fibroblasts electrically couples to cells of the amygdala or insula via connexin 43 gap junctions or indirectly via glial cells. This idea can provide an inherent “psyche” component for this rheumato-psycho-neurological mechanism. The relevance of this hypothesis to the works of scholars such as William Reich and Alexander Lowen and their essays on the bodily sensation of Self or muscular armoring and habitual muscular holding patterns requires further research in the field of psychobiology.

“Central sensitization,” even though not offering a satisfactory definite biological and cellular pathway to explain fibromyalgia (or to effectively tackle it pharmacologically), is to a certain degree limited to the (central) nervous system and to neuroendocrinology, while fascia is not. Also, unlike “central nervous system sensitization,” this theory can actually be investigated with direct measurements in living humans, as detailed in the following section (section 5.4), whereas functional magnetic resonance imaging does not measure hyperexcitability of neurons in the spinal cord dorsal horn or brain, and the term hyperalgesia is not interchangeable with “centralization of pain” or “central pain augmentation.” The former is part of the phenomenon, the latter relates to the theory. Although “central sensitization” is the prevailing paradigm for fibromyalgia, this theory primarily focuses on explaining augmented pain and hyperalgesia in the assumed absence of peripheral nociceptor activation (as it is, technically, a term borrowed from a spinal phenomenon described in pain studies in the 1990s in animal models) rather than the complex broad symptomatology and manifestations of the syndrome. For example, human assumed central sensitization doesn't seem to explain altering multisite deep aches instead of diffuse whole-body electric/burning pain, morning stiffness, itching, reduced skin innervation, multiple chemical sensitivity, muscle spasms, what are myofascial trigger points and tender spots and why their topography, poor response to theory-based pharmacological therapies, overlap and difference from chronic fatigue syndrome, evidence of functionally active autoantibodies, etc. In addition, the actual relevance of central sensitization animal models for fibromyalgia is unclear. In comparison to immune-based theories, the mechanical cascade of myofibroblasts is not necessarily propagated as an inflammatory disease in its nature and therefore can be mostly silent when examined in common medical investigations. Being triggered by surgery or trauma is only one of the several aetiologies of this model. The autoinflammatory repercussion of CD4^+^ T-cell dysregulation is ancillary. Systemic subclinical chronic oxidative stress is achieved by this theoretical model as it portrays a widespread mild-moderate chronic exertional compartment-like syndrome.

The neurobiological consequences of sustained nociception are similar to those of other chronic pain disorders, and include increased activity in the neuroendocrine and hypothalamic-pituitary-adrenal axes, response of the cortico-limbic and dopaminergic systems, potential neuroplastic changes, re-activation of microglia, upregulation and eventual exhaustion of endogenous anti-nociceptive inhibitory pathways, and more. Although the central nervous system was not focused on in this paper, humoral and neuroendocrine processes are deeply involved in “fascial armouring” and constitute a part of this entity.

### 5.4 Means for testing the hypotheses

Testing the model of fascial armouring can be done in several different ways.

• Biophysical tests-strain elastography, rheometry, stress-relaxation tests, biaxial tensile testing, atomic force microscopy, optical coherence elastography, compression tests, dynamic mechanical analysis, *etc.*, of fascial/myofascial tissue might be insightful, although these would have to take into account the complexity of the model and possible confounding factors, and control for hypermobility syndrome. Age, sex, pH, temperature, hydration, hyaluronic acid composition, adipocytes, cell phenotype and density, are all variables that may affect the properties of fascia *in vivo*.
• Optical Projection Tomography, Digital Light Sheet Microscopy, and Magnetic Resonance Microscopy can be used to study the 3D microstructure of fascia and its relationship to disease (Ugwoke et al., 2025). Analysis of fascia has also been done using cryogenic contrast-enhanced microcomputed tomography (Manon et al., 2024). Cryo-fixed histochemistry and low-vacuum scanning microscopy (Imazato et al., 2023) can help visualize three-dimensional ultrastructures of the tissue. The expected findings in fibromyalgia deep fascia would reflect diffuse ECM disarray, fibroblast proliferation and myofibroblast matrix remodeling and stress shielding. These may include altered collagen density and disorganization, abnormal collagen fiber orientation and tortuosity, 3D localization of fibrotic bands within specific fascial layers, altered fascial layer thickness, changes in overall tissue perimeter, gross collagen disorganization/densification, increased myofibroblast density, elongated fibroblast morphology, altered cell orientation, visible α -SMA stress fibers, and altered epimysial layer thickness corresponding with altered Young’s modulus.
• One straightforward non-invasive approach would be to use shear wave elastography or magnetic resonance elastography for more accuracy, which can help measure the stiffness of tissue to compare fibromyalgia patients and healthy controls. We would not necessarily expect the specific area with pain to be strictly correlated with stiffness because pain is the total sum of factors, starting from the nerve itself, the density of innervation, tethering or entrapment, inhibitory pathways, and all the way up to the brain and consciousness. We would, however, expect to find significant differences between patients and controls, and some correlation between imbalance in the overall bio-tensegrity structure and pain, and to find a temporal correlation between improvement in the overall stiffness/imbalance and improvement in pain, focusing on cases where other known pathologies have been excluded.
• The heterogenous nature of the syndrome is part of the mechanistic variability. When the biophysical matrix transformation of fascial tissue reinforces the cycle of myofibroblast force generation, myofascial stiffness progressively increases and muscles are subjected to low-grade chronic ischaemia. Over time, in the absence of full muscle relaxation, muscle mass and muscle cells under longstanding non-severe hypoxia will undergo long term structural, metabolic, and genetic, adaptations as part of the compensation, while the immune system is continuously drawn into the process by tissue injury. Sedentarism further reinforces skeletal muscle atrophy. Deposition of ECM material replaces atrophic muscle mass and then fatigue and weakness become more prominent. If fascia enters a stress-relaxation failure stage, where it experiences mechanical creep and has lower shear modulus, the bio-tensegrity system will be affected accordingly. Higher fascial stiffness and lower muscle tissue stiffness would have structural and functional consequences.
• Objective measurements of muscle damping (Wachter et al., 1996) should reflect increased muscle tension rather than spasticity. Pendulousness of the legs of patients compared to controls could be a relatively simple clinical test to start with.
• The structural and biochemical properties of fascia suggest it can behave similarly to a semiconductor or a biological capacitor to some degree as collagen fibers can conduct piezoelectric currents generated by mechanical stress and ground substance hyaluronic acid layers can contain negative charge. This should be evident in the electrical and magnetic properties of fascia of fibromyalgia patients in terms of electrical capacitance and more. The effect of weather changes on symptoms will be mediated by the effects of temperature, electromagnetics and humidity on myofascial tissue biophysical properties and on hyaluronic acid.
• Microdialysis of muscle, for example, of the trapezius muscle (Gerdle et al., 2020) or vastus lateralis (Gerdle et al., 2016): not all patients necessarily have increased concentrations of algesic substances and signs of anerobic metabolism in the same muscles because not all patients necessarily have the trapezius (or vastus lateralis) deeply affected. The clinical variability will be derived from a mechanistic variability regarding which areas and anatomical structures, and connective tissue layers, are more involved.
• Biopsies can be taken to examine whether there is an increase in myofibroblast density, or if cells express higher levels of smooth muscle actin, and to examine whether areas of higher density are associated with the location of pain and the severity of pain. Severe cases of fibromyalgia, for example, are expected to have significantly more myofibroblasts during the progressively worsening stage. But since myofibroblast can de-differentiate and leave behind a remodeled dysfunctional fascia, testing only by this method might actually be deceptive in certain cases. Percutaneous needle biopsy might be useful for estimating the density of myofibroblasts (Schleip et al., 2018) by staining the sample for known myofibroblast markers (Schleip et al., 2018; Sun et al., 2016; Xie et al., 2018).
• Invasive interventions are expected to affect PPOP and fibromyalgia, especially if operating on the path of the myofascial chains that are most relevant. Studying the effect of needling therapy, or tensegrity-based needling, to improve pain and myofascial stiffness as measured by shear wave or magnetic resonance elastography would be an interesting study to conduct for testing this theoretical model.
• Issue with systemic pharmacological therapies: A key player in the activation and maintenance of myofibroblasts is the cytokine TGF-β. At first glance, based on the myofibroblast theory, systemically targeting the TGF-β pathway pharmacologically (e.g., via inhibition of TGF-β ligand, TGF-β receptor kinase inhibitors, targeting upstream regulators and downstream effectors) seems to present a promising therapeutic strategy for fibromyalgia. However, a significant obstacle is in the multifunctional role of TGF-β in the body. TGF-β is essential for numerous physiological processes, including immune regulation, cell growth, differentiation, and tissue repair. Indiscriminate, systemic inhibition of TGF-β can lead to a wide range of severe, “on-target” adverse effects such as cardiovascular toxicity, immune dysregulation, and more. A “one-size-fits-all” approach that blocks all TGF-β activity is not a viable long-term solution. The reason pharmacological treatments require prudence is because systemic drugs would distribute in an imbalanced tensegrity structure equally throughout (depending on perfusion), which can be an inherent disadvantage in this case. According to this framework, systemic steroids are expected to indiscriminately modulate the tensegrity structure, supposedly arbitrarily leading to an exacerbation or relief, depending on the structure. More specifically, the success of some pharmacological agents that target myofibroblasts will depend on the balance between pro-survival signals (e.g., mechano-transducing signals) and pro-apoptotic signals.
• Overall, the cytoskeletal morphology and secretory profile of myo/fibroblasts should be altered correspondently, reflecting higher ECM rigidity. Classic systemic markers are not easy to make out because the mechanism is not endocrine or blood-mediated in the main, and there is no leukocyte-driven overt inflammation. Besides, this model has an inherent variability in terms of which anatomical structures are involved. If myofibroblasts normally utilize serotonin (Dolivo et al., 2018), a widespread induction of myofascial myofibroblasts should come at the expense of other organ systems that require tryptophan and its derivatives for normal function.
• Above a certain threshold of substrate stiffness, mechanosignaling would disrupt the intracellular balance between proliferative and apoptotic signals. This is expected to be reflected by gene expression and compensatory mechanisms of myofascial fibroblasts.
• Psychology: If a patient’s close circle of social support and treating physician regard symptoms as factitious, this will be associated with more anxiety, more suicidality, and patients might be described as more catastrophizing, i.e., extroverting the pain. In other words, if the treating physician easily dismisses clinical symptoms that impair patient’s activities of daily living, some patients might communicate in a more “catastrophizing” manner.

Box 3 gives examples of specific testable hypotheses to draw from the model. Box 4 offers specific practical study designs for exploratory studies. Principles from Koch’s postulates on causality can be used also. Falsification: according to Karl Popper, for a theory to be truly scientific, it must be falsifiable, meaning there must be conceivable empirical observations or experiments that can potentially falsify the theory. This fascia-based theory can be falsified in a study that quantifies fascial stiffness and elasticity across multiple body regions in a large sample size cohort of fibromyalgia patients and well-matched controls, if these studies consistently show no statistically significant difference in specific, predicted fascial mechanical and biophysical properties between fibromyalgia patients and controls, or if the observed differences do not correlate well with patient-reported symptom burden. If an intervention successfully normalizes the hypothesized fascial mechanical properties, but there is no statistically and clinically significant corresponding reduction in clinical outcomes compared to a credible sham or control intervention, this will weaken the causal link posited by the theory.

Box 3 **Examples of specific hypotheses to draw for future empirical studies.**

1. Myofibroblast phenotype cells are upregulated in myofascial tissue of fibromyalgia patients compared to normal individuals, and the matrix remodelling is reflected by such.
2. The expression of interleukin (IL) 6, IL-8 and IL-11 and alpha-smooth muscle actin will be significantly elevated in intrafascia cells of fibromyalgia patients compared to non-fibromyalgia controls, despite no clear overt inflammatory processes in a clinical examination. Values should correlate with severity of disease.
3. Severe fibromyalgia will be associated with signs of sustained low-grade muscle tissue ischaemia, such as blood creatine and lactate.
4. Muscle damping will be higher in all four limbs of fibromyalgia compared to age, sex, and BMI matched controls. Intramuscular pressure will be elevated not only in the trapezius muscle and despite absence of voluntary contraction and despite genuine attempts to relax.
5. Hypervigilance and recurrent worrying thoughts about pain in fibromyalgia, when occurring, are mainly the psychological consequence of ECM myofascial tissue abnormalities and should be significantly relieved with their correction, corresponding with a causal relationship.
6. In saliva and blood of fibromyalgia, cytokines and factors associated with myofibroblast induction and transforming growth factor beta-1 signaling will be positively correlated with fibromyalgia severity. These include connective tissue growth factor (CTGF), IL-6, IL-8, IL-17A, and IL-11 (depending on their half-life time).

Box 4 **Specific study designs for hypotheses.**

1. Obtain 3–5 mm punch biopsies of myofascial tissue from a standardized, accessible site (e.g., upper trapezius or lumbar paravertebral fascia) from both fibromyalgia patients and matched controls. Consider a washout period for relevant medications if clinically feasible. Generalized joint hypermobility syndrome is another factor to control for. Fixate and stain for myofibroblast markers such as alpha-smooth muscle actin. Primary outcome measures are density (cells/mm^2^) or proportion of tissue area stained for α-SMA positive myofibroblasts. Since multiple conformations of this protein exist, choosing proper staining is needed to avoid false negative results. Secondary outcome measures can be quantitative measures of collagen fiber density, orientation (anisotropy), collagen type I/III ratio, and expression/distribution of other ECM components. Statistical test for comparison of groups: independent samples t-test or Mann-Whitney U test (depending on data distribution) to compare myofibroblast density, ECM component quantification, and stiffness measures between fibromyalgia patients and controls. Correlation with matrix remodeling: Pearson’s or Spearman’s correlation coefficient to assess the relationship between myofibroblast density/markers and ECM remodeling parameters (e.g., collagen density, stiffness measures).
2. Can be combined with design of previous study to investigate gene expression (RT-qPCR or RNA-Seq): Isolate total RNA from myofascial tissue homogenates or isolated intrafascia cells. Quantify mRNA expression levels of IL-6, IL-8, IL-11, and ACTA2 relative to other housekeeping genes avoiding confounding that may be caused by increased tissue stiffness and subsequent mechano-sensitive signalling pathways. For protein expression (ELISA, Luminex, or Western blot) prepare protein lysates from tissue homogenates. Quantify protein levels of IL-6, IL-8, IL-11, and α-SMA using specific immunoassays. Alternatively, perform quantitative immunohistochemistry/immunofluorescence on tissue sections to assess protein expression and localization within fascial cells. Document normal C-reactive protein and erythrocyte sedimentation rate to confirm the absence of overt systemic inflammatory processes. Disease severity assessment can be done by administering validated fibromyalgia severity scales such as the Fibromyalgia Impact Questionnaire, Widespread Pain Index, and Symptom Severity Scale. Primary outcome measures are relative mRNA and/or absolute protein expression levels of IL-6, IL-8, IL-11, and α-SMA in myofascial tissue/cells. Secondary outcome measures can be scores on fibromyalgia severity scales. Statistical test for comparison of groups: independent samples t-test or Mann-Whitney U test to compare mRNA/protein expression levels between fibromyalgia and control groups. Correlation with severity can be done using Pearson’s or Spearman’s correlation coefficient to assess the strength and direction of the relationship between cytokine/α-SMA expression levels and fibromyalgia severity scores.
3. Cross-sectional study: fibromyalgia patients (potentially stratified into mild/moderate vs severe subgroups based on severity scores) and age-, sex-, and BMI-matched healthy controls. Collect fasting venous blood samples for lactate at baseline and potentially after a standardized, submaximal exercise challenge to assess metabolic recovery/capacity. Creatine kinase can be measured as a marker of general muscle cell distress or damage. Other systemic markers of ischemia/hypoxia: e.g., endothelin-1, adenosine, HIF-1α in plasma (though plasma levels might not reflect local tissue state well). Localized muscle microdialysis (invasive, but more specific): Perform microdialysis in a target muscle (e.g., trapezius, vastus lateralis) to directly collect interstitial fluid. Analyze for local concentrations of lactate, pyruvate, ATP, ADP, pH, nitric oxide, and other relevant biochemical factors. Non-invasive muscle perfusion assessment: Near-Infrared Spectroscopy: to continuously monitor local tissue oxygen saturation and changes in oxy-/deoxyhemoglobin during rest, standardized exercise (e.g., handgrip, calf raises), and recovery phases (e.g., vascular occlusion test). MRI protocol is available to non-invasively measure resting muscle perfusion. If done together with studies in points 1, 2, and 4, testing for correlation between hypoxic measures and matrix stiffness mechanical properties can be done.
4. Cross-sectional study using shear wave elastography or magnetic resonance elastography: To quantify the Young’s modulus (tissue stiffness) in multiple muscles across different body regions (e.g., trapezius, lumbar paraspinals, vastus lateralis) bilaterally. The sample size depends on the method. Intramuscular pressure measurement (invasive): use a pressure monitor inserted into target muscles (e.g., trapezius, vastus lateralis, potentially a lower limb muscle). Measurements are to be taken at rest, with explicit instructions for relaxation, and confirmation of absence of voluntary muscle contraction via concurrent surface electromyography. Patient-reported outcomes: visual analog scale or numerical rating scale for pain intensity, fibromyalgia impact questionnaire, and subjective ratings of muscle stiffness. Primary outcome measures are muscle stiffness mean values across all four limbs, and resting intramuscular pressure (in mmHg or kPa) in the trapezius and other selected muscles (measuring bilaterally). Statistical tests can include t-test or Mann-Whitney U test to compare resting intramuscular pressure in specific muscles between fibromyalgia and control groups. Pearson or Spearman correlation coefficients to assess the relationship between objective damping/pressure measures and self-reported pain/stiffness or disease burden as assessed by the fibromyalgia impact questionnaire.

## 6 Conclusion

The conceptual framework of bio-tensegrity may help understand and explain complex biological phenomena, from the level of the single cell to its microenvironment, the level of the tissue, organ, and up to the level of the whole organism–its behavior, function, kinematics, and kinesthetics, in health and disease. This manuscript explored a theoretical model and a mechanistic explanation for fibromyalgia-type syndromes that may also be relevant for PPOP, or least in those cases where other causes of pain have been excluded and the pain does not really “make much sense” clinically. The notion of a pathophysiological connection between fibromyalgia and PPOP is not new. Contemporary scientific literature frequently highlights the role of central sensitization in both conditions, suggesting a common underlying mechanism. This paper offers an alternative perspective, focusing on a peripheral mechanical mechanism. The interplay between abnormal extracellular matrix, a neuroma's intrinsic excitability, and peripheral and central neurophysiological compensatory mechanisms, collectively provide a neuropathophysiological basis to help explain PPOP. From the perspective of fascia, we may anticipate that invasive interventions will alter bio-tensegrity dynamics and will have non-negligible long-lasting effects on physiology and on pain because of myofascial chains and due to the interconnected nature of the pre-stressed bio-tensegral osteomyofascial system. Nociceptors are one of several types of structures embedded within fascia. Although the model focuses more on mechanobiology and biomechanics, the emotional and psychological aspects are no less important. Biology does not separate or segregate itself into demarcated sub-specialties like we do in our profession, and the “body” and “mind” are one Being, one flesh. The link between the central nervous system and fascia is a neglected field of research. This paper offered a theoretical model for PPOP (a condition which is often dismissed as a “non-disease” or invalidated as “pain catastrophizing”) in cases where no clear organic finding can be found to explain it (e.g., no nerve entrapment, nerve injury, radiation therapy, chemotherapy, *etc.*), and may open a new avenue for research on the link between neurobiology and connective-tissue. PPOP still poses a significant challenge to patients, clinicians, and researchers alike. Further research is needed to help us better understand the underlying mechanisms of persistent postsurgical pain.

## Data Availability

The original contributions presented in the study are included in the article/supplementary material, further inquiries can be directed to the corresponding author.
